# Barbed versus traditional sutures in primary total knee arthroplasty: systematic review and meta-analysis of clinical outcomes and wound-related complications

**DOI:** 10.1186/s13018-026-07036-3

**Published:** 2026-06-28

**Authors:** Hamed W. Babtain, Abdalmalik T. Malki, Awwab A. Shahhatalsayed, Basmah S. Alweal, Retal E. Faidah, Anas M. Alqarni, Loai A. Alsulaimani, Abdulrahman H. Albadri, Muath S. Alqahtani, Dhafer S. Almuffarh

**Affiliations:** 1 College of Medicine, King Saud bin Abdulaziz University for Health Sciences, Jeddah, Saudi Arabia; 2 King Abdullah International Medical Research Center (KAIMRC), Jeddah, Saudi Arabia; 3 Orthopedic Department, King Abdulaziz Medical City, Jeddah, Saudi Arabia

**Keywords:** Barbed suture, Traditional suture, Total knee arthroplasty, Infection, Closure time

## Abstract

**Background:**

Despite the widespread use of total knee arthroplasty (TKA), the optimal wound closure technique remains clinically unresolved. Barbed sutures have been proposed as a knotless alternative to traditional sutures, with potential advantages in operative efficiency and wound closure; however, concerns remain regarding postoperative complications. This systematic review and meta-analysis aimed to compare the clinical outcomes and postoperative complications associated with barbed versus traditional sutures in patients undergoing primary TKA.

**Methods:**

A comprehensive literature search was conducted using MEDLINE/PubMed, Cochrane Central Register of Controlled Trials (CENTRAL), ScienceDirect, Scopus, and Embase from database inception through November 2025. Eligible studies were restricted to randomized controlled trials (RCTs) involving adults undergoing primary TKA that directly compared barbed with traditional (knotted) sutures and reported at least one predefined clinical or wound-related outcome. Primary outcomes included wound closure time, superficial wound infection, postoperative Knee Society Score (KSS), knee range of motion (ROM), and wound-related adverse events. Meta-analysis was performed using RevMan version 5.3. The study was registered in PROSPERO (CRD420251128395) and received Institutional Review Board (IRB) approval from King Abdullah International Medical Research Center (Protocol No. NRJ25/044/8).

**Results:**

Five randomized controlled trials involving 1035 patients mettheeligibilitycriteria. Barbed sutures appeared to be associated with shorter wound closure times (MD = − 5.39 min, 95% CI: − 9.89 to − 0.88; *P* = 0.02). However, substantial heterogeneity was observed across studies. Superficial wound infection was significantly more frequent with barbed sutures (OR = 2.33, 95% CI: 1.07–5.05; *P* = 0.03). No significant differences were observed in postoperative KSS at 6 weeks and 3 months (MD = 0.42, 95% CI: − 0.88 to 1.72; *P* = 0.53).

**Conclusion:**

Barbed sutures may reduce wound closure time during TKA but are associated with a higher risk of superficial wound infection. No significant differences were observed in early functional outcomes, and further high-quality studies are needed to clarify long-term safety and efficacy.

## Introduction

Total knee arthroplasty (TKA) represents one of the most frequently performed orthopedic procedures worldwide, reflecting its effectiveness in managing advanced knee joint osteoarthritis [1]. One of the most important aspects in TKA is the wound closure as it has variable impacts on the operation time, wound healing, surgical site infection incidence, skin closure, and postoperative rehabilitation [2]. However, inadequate closure may result in wound complications such as infection, wound dehiscence, and hypertrophic scarring [3]. Recognizing suture-related wound issues is crucial for optimizing postoperative outcomes [3]. Therefore, appropriate closure techniques during the surgery help to prevent complications and increase the success of the procedure [4]. 

Traditionally, wound closure is done with interrupted knots with absorbable sutures in which are called the traditional suturing technique in this context [5]. However, one of the downsides of the traditional suturing is that it prolongs the operation time [6]. On the other hand, barbed suturing techniques referred to self-anchoring (barbed) sutures are designed with hooks along their length, either unidirectional or bidirectional, that grip tissue and hold it in place without the need for conventional knot tying to secure it [7]. Barbed suturing has been suggested to reduce operative time by eliminating the need for knot tying, giving the nature of the barbed suture self-locking mechanism [8]. 

Previous systematic reviews and meta- analyses have shown that the use of barbed sutures was significantly associated with reduced operative time and overall cost [8]. However, the current literature remains inconsistent regarding postoperative wound complications and functional outcomes in patients undergoing total knee arthroplasty, with several studies reporting conflicting findings [9, 10]. Additionally, previous evaluations failed to establish a clear consensus regarding the balance between operative efficiency and wound related complications associated with barbed sutures, particularly in TKA. Therefore, an updated systematic review and meta-analysis focusing specifically on TKA is needed to provide a more comprehensive evaluation of the comparative safety and effectiveness of barbed versus traditional suturing techniques.

This systematic review and meta-analysis aim to assess the outcomes and postoperative complications associated with barbed sutures versus traditional sutures in patients undergoing TKA in order to address the current uncertainty and lack of uniformity in assessing the reported outcomes of between both techniques.

## Methods

### Study design and search strategy

This meta-analysis began with a comprehensive literature search that follows PRISMA guidelines [11]. Two investigators independently performed the comprehensive search using MEDLINE/PubMed, Cochrane Central Register of Controlled Trials (CENTRAL), ScienceDirect, Scopus, and Embase from inception to 13 November 2025. The following Medial Subjective Headings (MeSH) and free words were used in the strategy (“barbed suture” OR “knotless suture” OR “self-retaining suture” OR “self-anchoring suture” OR “Quill” OR “V-Loc” OR “Stratafix”) AND (“traditional suture” OR “conventional suture” OR “smooth suture” OR “non-barbed suture” OR “knotted suture” OR “standard suture”) AND (“total knee arthroplasty” OR “TKA” OR “total knee replacement”). After database screening, reference lists of all included trials were manually checked for additional eligible studies. No hand-searching of specific journals and no expert consultation were undertaken, consistent with the review protocol. This study was registered with the International Prospective Register of Systematic Reviews (ID: CRD420251128395) and received Institutional Review Board (IRB) approval from King Abdullah International Medical Research Center (Protocol No. NRJ25/044/8).

### Eligibility criteria

The eligibility criteria were defined using the PICOS framework and were restricted to randomized controlled trials only as stated in the protocol. Studies were included when they fulfilled the following criteria: (1) randomized controlled trials (2) adults ≥ 18 years undergoing primary total knee arthroplasty (3) studies included wound closure using barbed sutures versus Traditional (knotted) suture. (4) studies in English (5) reporting at least one predefined clinical or wound-related outcome. The predefined primary outcomes were wound closure time, superficial wound infection, Knee Society Score (KSS) postoperatively, needle disengagement, knee range of motion (ROM), and other wound-related adverse events including wound dehiscence, stitch abscess, and deep wound infection. Secondary outcomes included hospital stay and patient satisfaction. Exclusion criteria were: (1) revision and bilateral Total knee Arthroplasty (2) total knee Arthroplasty combined with other procedures (3) non-Randomized-controlled trials and non-clinical studies. (4) studies evaluating isolated closure of a specific tissue layer (e.g., capsular closure only) rather than complete wound closure techniques (5) studies using modified or proprietary wound closure systems.

### Data extraction

Data extraction was performed independently by two reviewers using a standardized form. Extracted variables included the study author, year of publication, country, sample size, patient demographics (age, gender, and BMI), follow-up duration, key reported outcomes, and complications. Any discrepancies between the two reviewers were resolved through discussion and consultation with a third independent reviewer who was not involved in the initial screening or data extraction process. When essential information was missing or unclear, attempts were made to contact the study authors to obtain the required data.

### Quality assessment

The methodological quality of the included studies was evaluated using the Cochrane Risk of Bias 2 (RoB 2) tool for randomized controlled trials [12]. Two reviewers independently assessed each study across the five RoB 2 domains: (1) bias arising from the randomization process, (2) bias due to deviations from intended interventions, (3) bias due to missing outcome data, (4) bias in measurement of the outcome, and (5) bias in selection of the reported result. For each domain, signaling questions were answered according to the RoB 2 guidance, and domain-level judgments were categorized as low risk, some concerns, or high risk of bias based on the algorithm-generated results and reviewer interpretation. Any disagreements were resolved by a third reviewer. Overall risk of bias for each study was derived according to the RoB 2 decision rules.

### Data analysis

For continuous outcomes, effect sizes were calculated using the mean difference (MD) with corresponding 95% confidence intervals (CI). For categorical outcomes, odds ratios (ORs) with corresponding 95% confidence intervals (CI) were calculated. Statistical heterogeneity among studies was assessed using the chi-square (Q) test and the I^2^ statistic. A fixed-effect model was applied when heterogeneity was low (I^2^ ≤ 50% and *P* > 0.10), whereas a random-effects model was used when substantial heterogeneity was present (I^2^ > 50% and *P* < 0.10). Sensitivity analyses were conducted by excluding studies judged to be at high risk of bias. Meta-analysis was performed using RevMan version 5.3, and forest plots were generated for outcomes reported in at least two studies. When applicable, subgroup analyses were undertaken to compare different closure types (e.g., barbed vs. running sutures, staples vs. sutures, and adhesive techniques).

## Results

### Study selection

**Fig. 1:**
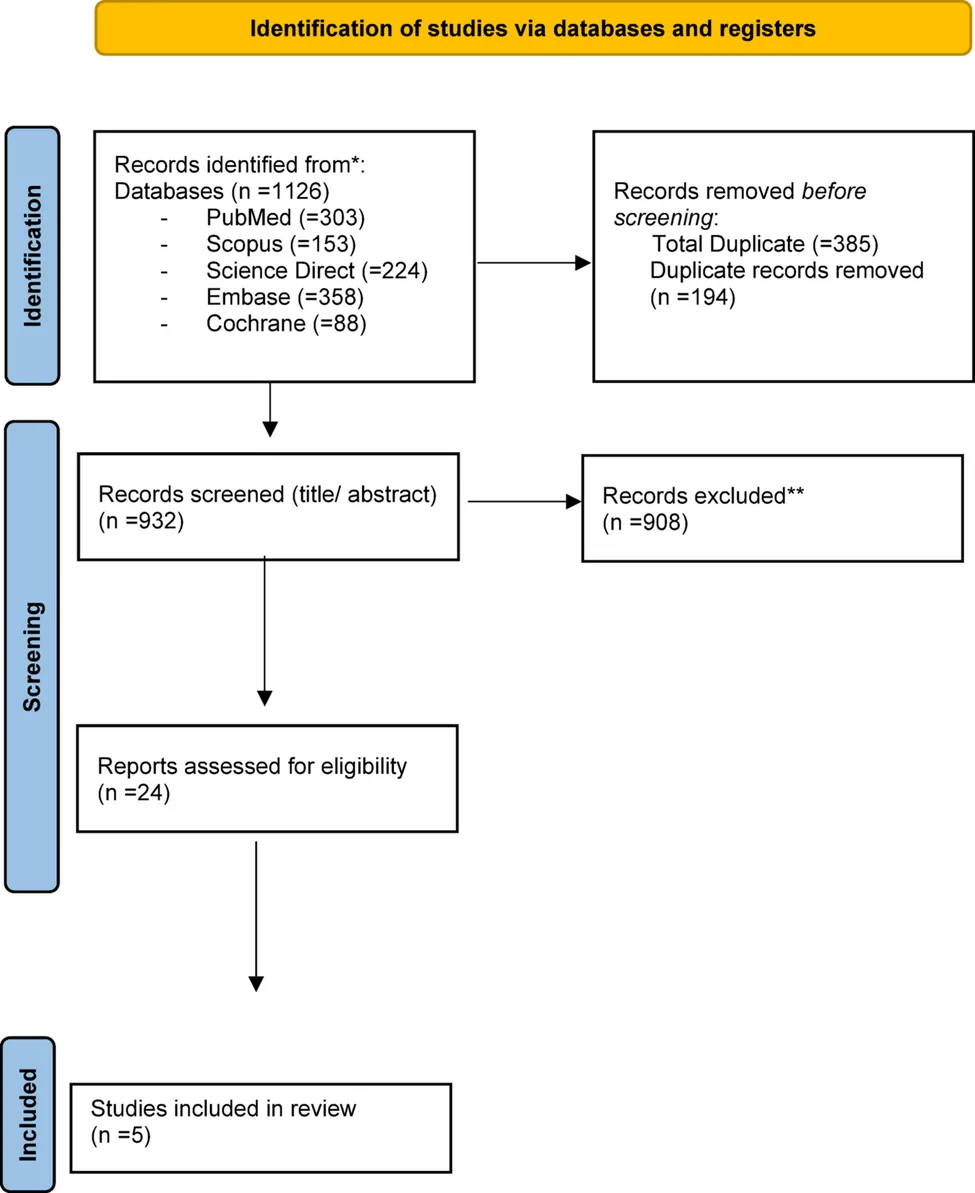
Flowchart of study inclusion and exclusion conducted under the Preferred Reporting Items for Systematic Reviews and Meta-Analysis (PRISMA) guidelines

A total of 1126 articles were identified through MEDLINE/PubMed, Cochrane Central Register of Controlled Trials (CENTRAL), ScienceDirect, Scopus, and Embase. Following removal of 194 duplicates, 932 articles underwent title and abstract screening. Of these, 24 studies were assessed in full text, and five randomized controlled trials met the inclusion and exclusion criteria were ultimately included in the review (Fig. 1).

### Characteristics of the included studies

The review included 5 randomized controlled trials from 2014 to 2020 with total of 1035 patients who underwent primary total knee arthroplasty. 510 (49.28%) of the patients received wound closure using barbed sutures where the other 525 (50.72%) received traditional sutures. The mean age was comparable between the groups, with averages of 67.66 years in barbed suture group and 67.58 years in the traditional suture group. Female gender showed dominance across both types of suturing with 70.84% of the participants in barbed group and 73.88% in traditional suturing group (Table 1). Further methodological details on wound closure techniques are presented in Table 2. Notable variability was observed among the included randomized controlled trials regarding the layers in which barbed sutures were applied, the materials used for skin closure, and the reporting of perioperative antibiotic prophylaxis. In some studies, barbed sutures were limited to the arthrotomy or capsular layer, while in others they were used across all wound layers, including subcutaneous and intracutaneous or subcuticular closure. Skin closure approaches differed between studies and included metallic staples, absorbable subcuticular sutures, Dermabond, and surgeon-preferred techniques. Reporting of antibiotic prophylaxis was inconsistent, with only one study clearly outlining a standardized perioperative regimen (Table 2).

**Table 1:** Characteristics of the Included studies in this SR-MA

Study	Country	Sample size	Total barbed suture *n*(%)	Total traditional suture *n*(%)	Follow up period post-surgery	Female gender *n*(%)	Age (years) mean (± SD)	BMI mean (± SD)	Clinical outcomes reported
Gililland et al. [13]	US	394	191 (48.48%)	203 (51.52%)	Up to 6 weeks	Barbed: 114 (60%) Traditional: 126 (62%)	Barbed: 64 (± 10) Traditional: 63 (± 10)	Barbed: 33 (± 8) Traditional: 33 (± 8)	Wound closure time, cost, perioperative complications, KSS, patient satisfaction, cosmetic outcomes, wound infection outcomes.
Gamba et al. [15 ]	Spain	85	41 (48.24%)	44 (51.76%)	1 month	Barbed: NA (61.4%) Traditional: NA (68.3%)	Barbed: 74.2 (± 8.2) Traditional: 73.8 (± 7.5)	Barbed: 30.2 (± 5) Traditional: 30.6 (± 4.6)	Wound closure time (capsular, subcutaneous, total), ROM, superficial infection, deep infection, wound dehiscence, intraoperative suture breakage.
Sakdinakiattikoon et al. [20]	Thailand	60	30 (50%)	30 (50%)	3 months	Barbed: 23 (76.7%) Traditional: 25 (83.3%)	Barbed: 64.8 (NA) Traditional: 65.7 (NA)	Barbed: 64 (NA) Traditional: 63 (NA)	Wound closure time, KSS, cost, superficial infection, deep infection, wound dehiscence, and postoperative drainage
Feng et al. [16]	China	387	193 (49.87%)	194 (50.13%)	3 months	Barbed: 140 (72.5%) Traditional: (140) 72.2%	Barbed: 64.8 (± 8.7) Traditional: 65 (± 8.7)	Barbed: 24.1 (± 2.7) Traditional: 24 (± 2.7)	Wound complications (effusion, superficial and deep infection, hematoma, skin necrosis, blisters), wound closure time, operation time, hospital stay, KSS.
Chan et al. [14]	China	109	55 (50.46%)	54 (49.54%)	Up to 3 months	Barbed: 46 (83.63%) Traditional: 47 (83.63%)	Barbed: 70.5 (± 8.2) Traditional: 70.4 (± 8.9)	Barbed: 26.8 (± 1.2) Traditional: 26.5 (± 3.9)	Wound closure time, cost analysis, ROM, (KSS), superficial and deep infection, stitch abscess, superficial cellulitis, superficial wound dehiscence, leak test results, intraoperative events

**Table 2:** Wound closure characteristics, skin closure methods, and antibiotic prophylaxis across included randomized controlled trials

Study	Barbed suture used in which layers	Traditional/Control closure	Skin closure material	Antibiotic prophylaxis reported
Chan et al. [14]	Arthrotomy + subcutaneous layers using Stratafix barbed sutures	Arthrotomy: interrupted Vicryl; subcutaneous: continuous Vicryl	Staples (Appose, Medtronic) in both groups	Not clearly reported
Sakdinakiattikoon et al. [20]	Capsular + subcutaneous + subcuticular skin closure with barbed sutures	Capsular + subcutaneous interrupted Vicryl; skin with running subcuticular Vicryl	Barbed group: Monoderm barbed subcuticular closure; traditional group: Vicryl subcuticular closure	Not specifically reported
Feng et al. [16]	Group A: capsule + subcutaneous + intracutaneous; Group B: capsule only	Group B: subcutaneously. Group C: capsule + subcutaneous absorbable sutures	Groups B/C: staples; Group A: intracutaneous barbed closure	Yes, single IV prophylactic dose before surgery and continued for 24 h
Gililland et al. [13]	Arthrotomy + subdermal layers with running barbed sutures	Arthrotomy: interrupted Ethibond; subdermal: interrupted Monocryl	Variable; surgeon preference (staples, steri-strips, Dermabond, subdermal sutures)	Not reported
Gamba et al. [15]	Capsular + subcutaneous layers with Quill barbed sutures	Vicryl interrupted sutures for both layers	Metallic staples in all cases	Not reported

### Risk of bias

**Fig. 2:**
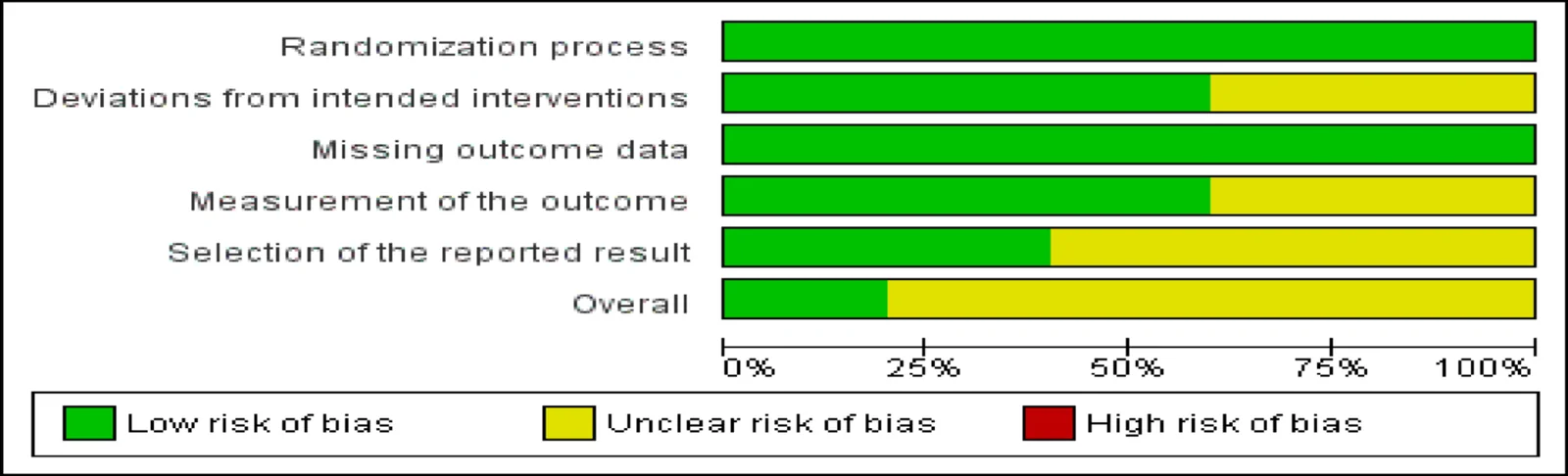
Risk of bias assessment graph for randomized controlled trials.

**Fig. 3:**
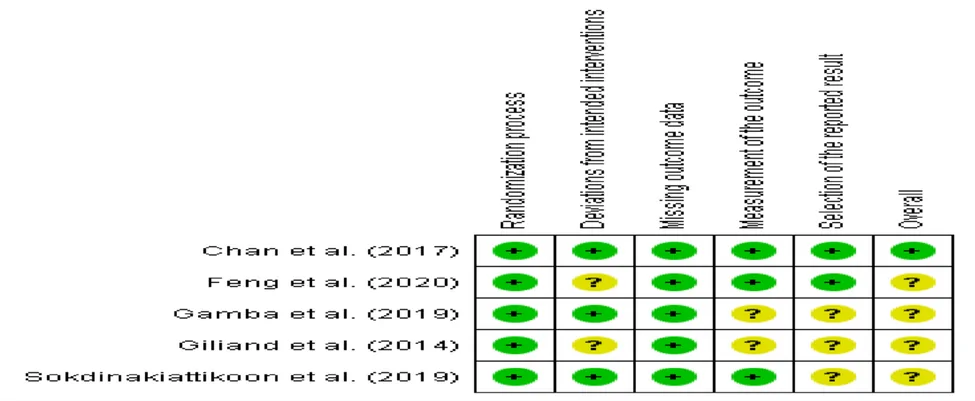
Risk of bias summary for randomized controlled trials

Risk of bias for all included RCTs was evaluated using the Cochrane Risk of Bias tool (RoB2) (Figs. 2 and 3**)**. All the studies except Gililland et al. [13] showed some concerns in the overall risk of bias rating. Specifically, while all trials adequately reported data completeness and followed appropriate randomization procedures, more than half exhibited concerns related to selection bias, deviations from intended interventions, and bias in outcome measurement. Notably, Chan et al. [14] demonstrated the highest risk of bias as it reported some concerns in 3 domains including deviation from intended results, selection bias as well as bias in outcomes’ measurements.

### Outcome analysis

#### Wound closure time

**Fig. 4:**
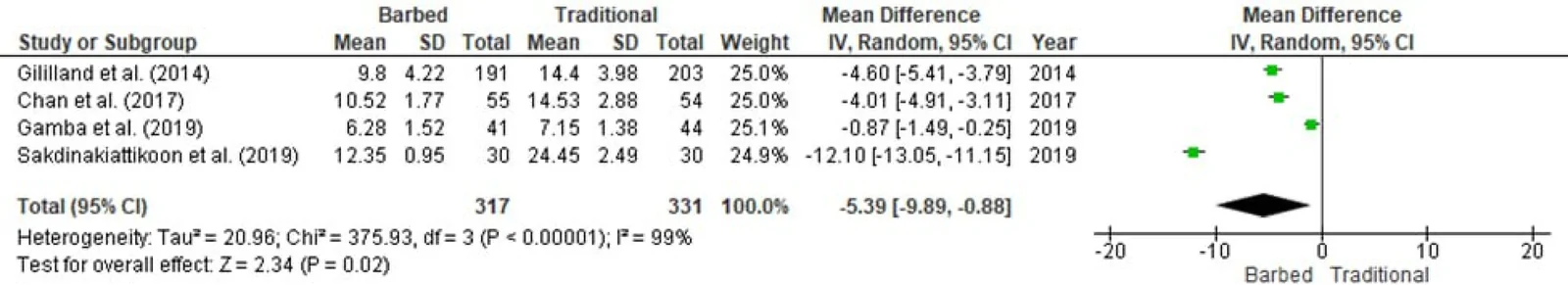
Meta-analysis of time for wound closure using barbed versus traditional sutures

Out of all studies included in this systematic review and meta-analysis, four of them reported wound closure time. The total number of patients is 648 out of which 317 were in the barbed arm. All the analysed studies showed significant reduction of closure time in the barbed group compared to traditional group. The pooled estimates demonstrated a statistically significant reduction of closure time (MD = − 5.39, 95% CI: − 9.89 to − 0.88, *p* = 0.02). However, the heterogeneity is substantially high among the included studies (*p* < 0.00001, I^2^ = 99%), which suggests that there is considerable variability of effect size across the studies (Fig. 4).

#### Superficial wound infection

**Fig. 5:**
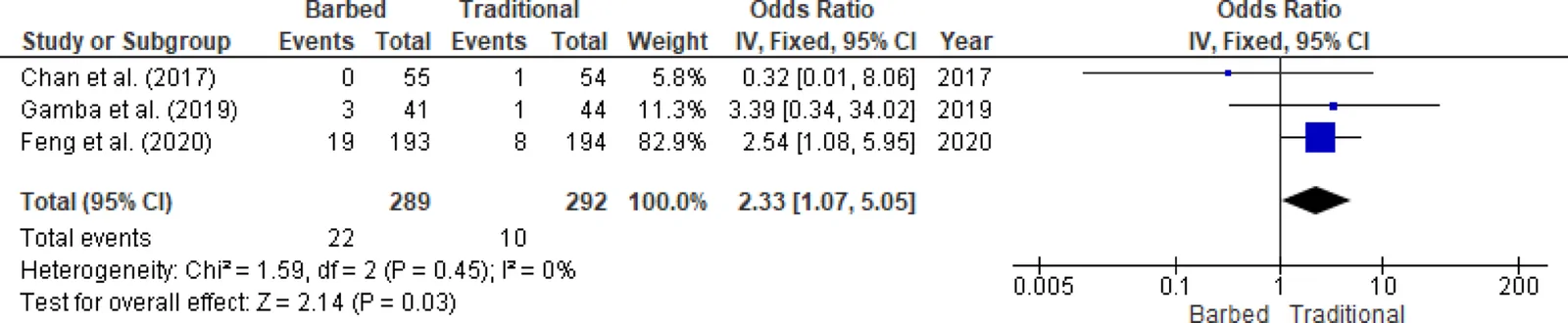
Meta-analysis of superficial wound infection using barbed versus traditional sutures

There were three studies that reported superficial wound infection after total knee arthroplasty. Overall pooled analysis demonstrated that there was a significant high risk of developing superficial wound infection in barbed suturing technique with a pooled odds ratio of 2.33 (95% CI: 1.07 to 5.05, *p* = 0.03). No heterogeneity was reported among the studies included (*p* = 0.45, I^2^ = 0%) which indicate a highly consistent result (Fig. 5).

#### 6 weeks post-operative knee society score

**Fig. 6:**
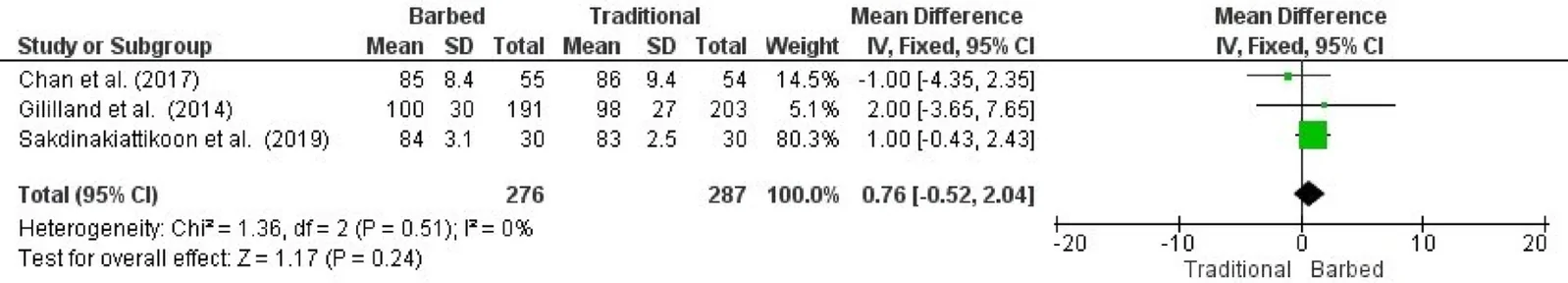
Meta-analysis of 6 weeks post-operative knee society score using barbed versus traditional sutures

At 6 weeks post-operative knee society score were only reported by 3 studies. There is a minor advantage in barbed suturing group; however, it’s not significant with (MD 0.76, 95% CI: − 0.52 to 2.04, *p* = 0.24). No heterogeneity was reported among the studies included (*p* = 0.51, I^2^ = 0%) which reflect a highly consistent result (Fig. 6).

#### 3 months post-operative knee society score

**Fig. 7:**
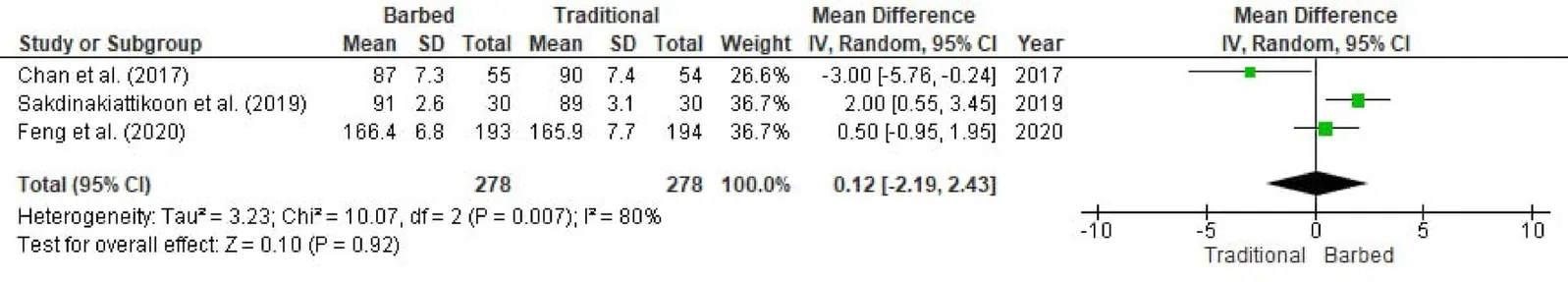
Meta-analysis of 3 months post-operative knee society score using barbed versus traditional sutures

At 3 months, knee society scores were compared between barbed and traditional sutures in three studies with an overall 556 patients out of them 278 use barbed suture. One study favored the traditional group, another study favored the barbed group, and one reported no significant difference. The overall pooled results of the meta-analysis indicate that there is no statistically significant difference between barbed and traditional sutures in knee society scores at 3 months (MD = 0.12, 95% CI: − 2.19 to 2.43, *p =* 0.92). However, heterogeneity was high between the 3 reported studies (*p* = 0.007, I^2^ = 80%) (Fig. 7).

#### 6 weeks and 3 months post-operative knee society score together

**Fig. 8:**
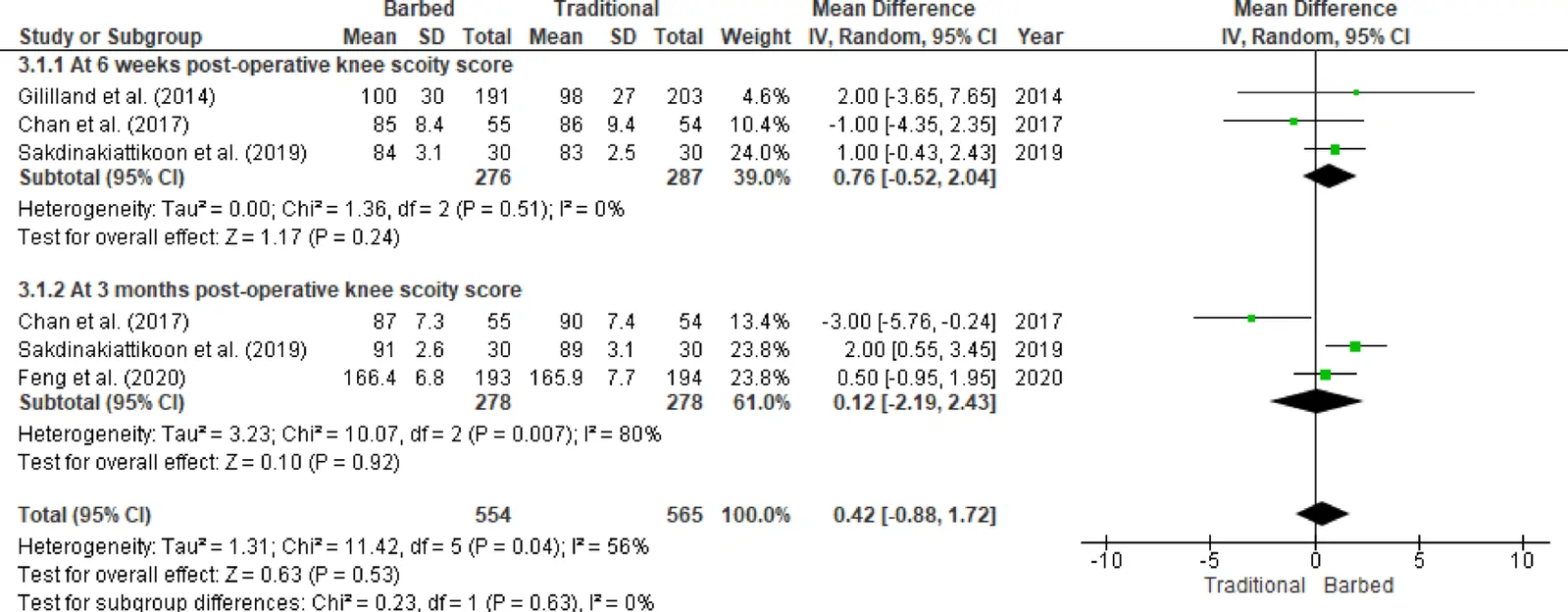
Meta-analysis of 6 weeks and 3 months post-operative knee society score together using barbed versus traditional sutures

Subgroup analysis based on duration of the follow-ups showed that there is no statistical difference between barbed and traditional sutures regarding the knee society score at either time point. The overall pooled results of the meta-analysis indicate that there is no statistically significant difference in knee society scores between the two techniques (MD = 0.42, 95% CI: − 0.88 to 1.72, *P* = 0.53). However, heterogeneity was high between the reported studies (*p* = 0.04, I^2^ = 56%) (Fig. 8).

#### Wound dehiscence

It was reported by only two studies [14, 15]. Chan et al. [14] mentioned that there is only 1 case with barbed suture among 55 patients compared to 3 cases of wound dehiscence among 54 patients with traditional group. However, Gamba et al. [15] reported that there are two events among 41 with barbed sutures and only one event among 44 patients with traditional techniques.

#### Needle disengagement

Also, it was reported in 2 studies only [13, 15]. Gamba et al. [15] mentioned that 17 events among 41 patients in barbed group and Gililand et al. [13] reported 12 events among 191 patients in barbed group also. However, both studies mentioned that there were no events of needle disengagement associated with traditional groups.

#### Stitch abscess

Stitch abscess was reported in 2 studies [13, 14]. Chan et al. [14] reported one case of stitch abscess in barbed suture and five cases in traditional group. However, Gililand et al. [13] reported that there were four events of stitch abscess in barbed suture and two events in traditional group.

#### Post-operative range of motion

Only two studies reported post operative range of motion [14, 15]. Chan et al. [14] and Gamba et al. [15] reported a mean postoperative range of motion of 101° ± 14.5 and 100.9° ± 5.93 in the barbed suture group respectively. However, it was 103° ± 12.1 and 102.4° ± 12.41 in the traditional suture group respectively.

#### Deep wound infection

Only two studies reported deep wound infection [15, 16]. Gamba et al. [15] mentioned that there were no cases in both barbed suture and traditional suture groups identified after total knee arthroplasty. Feng et al. [16] reported 4 cases of deep wound infection in barbed suture group compared to only two cases in traditional group.

## Discussion

This systematic review and meta-analysis compared traditional suturing techniques with barbed suturing techniques in patients undergoing TKA, including 1035 patients across RCTs. Overall, neither suturing technique demonstrated clear superiority across most measured clinical outcomes. However, our findings suggested a reduction in wound closure time favoring the barbed suturing group. These results are consistent with previous systematic reviews and meta-analyses. Raja et al. [17] reported a significant reduction in wound closure time of − 5.94 min in their meta-analysis of 16 RCTs. The discrepancy in the number of the included studies in this systematic review compared to Raja et al. [17] may be explained by the differences in the inclusion criteria, one example of difference between the two reviews is that in this study only RCTs of adult patients underwent total knee arthroplasty, whereas in Raja et al. [17] some of the included studies include both total knee and total hip arthroplasty. Similarly, Xin et al. [18] and Li et al. [19] reported significant reductions of − 4.895 min and − 4.41 min, respectively, associated with barbed sutures. Despite this consistent direction of effect, substantial heterogeneity was observed in our analysis (I^2^ = 99%), indicating considerable variability in effect sizes and limiting confidence in the magnitude and generalizability of the observed benefit. The heterogeneity was due to differences across studies, such as variations in the definition of wound closure time, the number of closure layers included skin closure methods, surgeon experience, and suture characteristics. Although all studies generally favored barbed sutures, the magnitude of reduction varied substantially, ranging from 0.87 min in Gamba et al. [15] to 12.10 min in Sakdinakiattikoon et al. [20] Therefore, the pooled estimate should be interpreted with caution.

In contrast, pooled analysis demonstrated a statistically significant higher risk of superficial wound infection associated with barbed sutures, with no observed heterogeneity (I^2^ = 0%), suggesting consistent findings across the included trials. However, these results are not consistent with those of Li et al. [21] who reported no statistically significant difference in superficial wound infection rates between barbed and conventional sutures. The differences in methodology and eligibility criteria between the two studies may explain this discrepancy. This systematic review and meta-analysis included only RCTs which involved adults who underwent unilateral primary TKA, while the previous review incorporated both RCTs and retrospective studies. Additionally, the previous review included larger sample size and greater number of studies which may increase the statistical power and affect the pooled estimates. These methodological differences may contribute to the difference in findings between both studies.

Regarding functional recovery outcomes, appropriate wound closure following TKA is essential to promote optimal joint rehabilitation. Both KSS and knee ROM are time-dependent measures that may vary throughout the postoperative course. Accordingly, this study conducted separate analyses of KSS at 6 weeks and 3 months postoperatively, whereas only one postoperative time point was available for ROM analysis. Our findings demonstrated non-significant difference in KSS between barbed and traditional sutures at 6 weeks, consistent with the findings of Li et al. [19] However, at 3 months postoperatively, our analysis showed no significant difference, whereas their systematic review reported superior outcomes with barbed sutures. These conflicting findings may be attributable to variations in included trials, follow-up duration, patient characteristics, rehabilitation protocols, or analytical approaches.

Several predefined primary outcomes including knee ROM, needle disengagement, deep wound infection, wound dehiscence, stitch abscess, and other wound-related complications could not be robustly evaluated because less than 3 studies reported these outcomes, thus, limited and reduced reliability and precision of pooled estimates and precluded meaningful assessment. An important parameter after total knee arthroplasty is knee range of motion (ROM) as it affects both postoperative functional recovery and rehabilitation [22]. However, insufficient reporting made it difficult to determine impact of suturing techniques on mobility outcomes. Also, Needle disengagement is a crucial technical outcome, as it affects the operative efficiency and surgeon handling during wound closure. Nevertheless, there is limited reporting regarding this outcome. Similarly, deep wound infection, wound dehiscence, and stitch abscess are clinically significant complications that may adversely affect the recovery and overall quality of life of such a patient. Future studies with greater sample sizes and more consistent reporting of relevant outcomes are needed to strengthen the evidence comparing barbed and traditional sutures. Future randomized controlled trials should incorporate standardized outcome definitions, clearly predefined primary and secondary endpoints, and adequate follow-up periods to enhance comparability across studies which will enable more robust pooled analyses.

Despite that barbed suture associated with multiple theoretical advantages such as uniform tension distribution and elimination of knot tying, their overall clinical benefit in TKA remains uncertain. Although they consistently demonstrate reduced wound closure time, this efficiency advantage must be carefully balanced against the observed increased risk of superficial wound infection and the limited evidence for several clinically relevant endpoints. Furthermore, a retrospective cohort study by Thacher et al. [23] reported a significantly increased risk of wound dehiscence associated with barbed sutures in arthroplasty patients. However, given its retrospective design and inherent susceptibility to selection bias and confounding, its level of evidence is lower than that of the RCTs included in the present analysis.

This study provides important insight into the role of barbed sutures in wound closure following TKA. It has several notable strengths. It included only randomized controlled trials, which improve the overall quality of evidence and help limit selection bias. The analysis was also restricted to patients undergoing unilateral primary total knee arthroplasty, reducing variability associated with different types of arthroplasty procedures. In addition, study selection, data extraction, and quality assessment were carried out independently by two reviewers using predefined criteria, with any disagreements resolved through third reviewer, thereby strengthening methodological rigor and minimizing the risk of reviewer bias. Nevertheless, the relatively small number of included RCTs, substantial heterogeneity observed for wound closure time, and limited reporting of several clinically important outcomes restrict the strength of the conclusions.

Collectively, although barbed sutures showed a significant reduction in wound closure time, their potential association with increased risk of superficial wound infections and insufficient evidence regarding other key outcomes underscores the need for further well-designed, sufficiently powered RCTs with standardized outcome definitions and longer follow-up durations to surely evaluate definitive conclusions regarding the safety and clinical efficacy of barbed sutures in total knee arthroplasty.

## Limitations

Several limitations of this study should be acknowledged. First, differences in outcome definitions and reporting across studies limited the ability to perform quantitative synthesis and contributed to outcome-reporting heterogeneity, precluding meta-analysis for several endpoints. Second, studies that did not provide separate data for barbed and traditional suture groups were excluded, potentially reducing the number of eligible trials. Third, economic analysis, including total cost comparisons, was not addressed in this review. Fourth, one of the major limitations of this review was the substantial heterogeneity observed in the wound closure time analysis (I^2^ = 99%). Sensitivity analyses were done by removing first Gamba et al. [15] and secondly by removing Sakdinakiattikoon et al. [20] However, neither one substantially reduced heterogeneity, which may suggest that the variability was not driven by a single study. Finally, the studies included in this systematic review predominantly reported short-term postoperative outcomes, limiting the ability to draw conclusions regarding long-term clinical effects of barbed versus traditional sutures in total knee arthroplasty.

## Conclusion

Barbed sutures in total knee arthroplasty appear to be associated with shorter wound closure times compared with conventional suturing techniques. However, the magnitude of this effect could not be reliably estimated because of the extreme heterogeneity between studies. Despite the potential operative efficiency advantage, barbed sutures were also associated with a significantly increased risk of superficial wound infection, a finding that was consistent across studies. Taken together, the current evidence suggests that any potential time saving benefit should be interpreted cautiously and weighed against the possibility of wound-related complications when selecting closure techniques in TKA.

Importantly, the available evidence does not demonstrate clear superiority of barbed sutures in terms of short-term functional recovery or other clinically relevant postoperative outcomes. Therefore, the principal clinical implication of this review is that the theoretical efficiency advantages of barbed sutures may not necessarily translate into improved overall patient outcomes, while concerns regarding superficial wound complications remain relevant.

The strength of these conclusions is limited by substantial heterogeneity in operative time outcomes, variability in study methodology and suture techniques, and the relatively limited evidence available for several postoperative complications. Further adequately powered, high-quality randomized controlled trials with standardized outcome reporting and longer follow-up are required before definitive recommendations regarding the routine use of barbed sutures in total knee arthroplasty can be made.

## Data Availability

All data generated or analyzed during this study are included in this published article and its supplementary materials. Additional data may be made available from the corresponding author upon reasonable request.

## Abbreviations

BMI: Body mass index
CENTRAL: Cochrane central register of controlled trials
CI: Confidence interval
IRB: Institutional review board
KAIMRC: King Abdullah International Medical Research Center
KSS: Knee society score
MD: Mean difference
MeSH: Medical Subject Headings
OA: Osteoarthritis
OR: Odds ratio
PRISMA: Preferred reporting items for systematic reviews and meta-analyses
RCT: Randomized controlled trial
RoB 2: Risk of bias 2 tool
ROM: Range of motion
SD: Standard deviation
TKA: Total knee arthroplasty
